# Real-World Setting Cost-Effectiveness Analysis Comparing Three Therapeutic Schemes of One-Year Adjuvant Trastuzumab in HER2-Positive Early Breast Cancer from the Cyprus NHS Payer Perspective

**DOI:** 10.3390/ijerph17124339

**Published:** 2020-06-17

**Authors:** Savvas S. Ioannou, Yiola Marcou, Eleni Kakouri, Michael A. Talias

**Affiliations:** 1 Healthcare Management Postgraduate Program, Open University Cyprus, P.O. Box 12794, Nicosia 2255, Cyprus; savvas.ioannou@st.ouc.ac.cy; 2 Department of Medical Oncology, Bank of Cyprus Oncology Center, 32 Acropoleos Avenue, 2006 Strovolos, Nicosia 2255, Cyprus; yiola.marcou@bococ.org.cy (Y.M.); eleni.kakouri@bococ.org.cy (E.K.)

**Keywords:** real-world data, real-world evidence, breast cancer, trastuzumab, oncology, QALYs, Markov model, cost-effectiveness analysis, data analysis in pharmaceutical therapy

## Abstract

Introduction: This study is one of the first real-world cost-effectiveness analyses of one-year adjuvant trastuzumab used in HER2-positive early female breast cancer in comparison to chemotherapy alone. It is just the second one in Europe, the first one in Cyprus, and the fourth one worldwide ever carried out using real-world data. Methods: Using a Markov model (four health states), a cost-effectiveness analysis was carried out both over 20 years and for a lifetime horizon. The sampling method used in this study was the randomized sampling of 900 women. Results: The findings for the 20-year horizon showed that all trastuzumab arms were more cost-effective, with a willingness-to-pay threshold of only €60,000 per quality-adjusted life year (QALY) [incremental cost-effectiveness ratios (ICER): €40,436.10/QALY]. For the lifetime horizon, with thresholds of €20,000, €40,000, and €60,000/QALY, all trastuzumab arms were found to be more cost-effective (ICER: €17,753.85/QALY). Moreover, for the 20-year and the lifetime horizons, with thresholds of €20,000/QALY, €40,000/QALY, and €60,000/QALY, the most cost-effective of the three subgroups (anthracyclines and then trastuzumab, no anthracyclines and then trastuzumab, and anthracyclines, taxanes, and trastuzumab) was that of anthracyclines and then trastuzumab (ICER: €18,301.55/QALY and €8954.97/QALY, respectively). Conclusions: The study revealed that adjuvant trastuzumab for one year in female HER2-positive early breast cancer can be considered cost-effective.

## 1. Introduction

Breast cancer had the highest incidence of all cancers in Cyprus for the 11 years between 1998 and 2008 [1]. In 2015, about 95,300 people died of breast cancer in the European Union (EU-28), of whom fewer than a thousand were men and the vast majority (94,300) were women [2]. As a result, breast cancer deaths account for 7.2% of all cancer deaths. Among women, breast cancer accounts for 15.6% of all cancer deaths [2]. Overexpression of the human epidermal growth factor receptor 2 (HER2) is present in approximately 17% of breast cancer patients and has been associated with aggressive tumor behavior, decreased response to traditional therapies, and reduced survival [3,4].

Several studies have shown that the use of adjuvant trastuzumab for one year in early HER2-positive breast cancer significantly improves the rate of survival without progression of the disease and reduces the risk of death [5,6,7,8]. Hence, the administration of adjuvant trastuzumab for one year in early HER2-positive breast cancer is considered a milestone in breast cancer therapy. To our knowledge, from 2007 to 2019, twenty cost-effectiveness surveys of one-year adjuvant trastuzumab in cases of early HER2-positive breast cancer were published worldwide [9,10,11,12,13,14,15,16,17,18,19,20,21,22,23,24,25,26,27,28]. Only three studies were published from 2007 to 2019 using real-world data: in Canada (2012), The Netherlands (2017), and Taiwan (2019) [19,21,26]. All the other investigations used data from clinical trials. Scholars have not yet reached a broad consensus about the cost-effectiveness of the use of adjuvant trastuzumab in early HER2-positive breast cancer. Several studies have concluded that the use of trastuzumab compared to conventional chemotherapy treatment was cost-effective and found favorable incremental cost-effectiveness ratios (ICERs) [11,12,14,17,19,21,22,23,24,25,26,27]. In contrast, other studies have reported that the use of trastuzumab was not cost-effective [9,10,13,15,16]. Furthermore, yet other studies have concluded that the cost-effectiveness of adjuvant trastuzumab remains unclear [18,20,28]. Most of the previous studies were performed from a healthcare provider perspective. The majority of them used Markov models and lifetime horizons [9,10,12,14,15,16,17,18,19,20,21,22,23,24,26,28]. Two systematic reviews revealed that most researchers used QALYs as the health outcome measurements to evaluate trastuzumab’s cost-effectiveness [29,30].

Real-world studies may be liable to selection bias, but they make available an accurate representation of the patient population found in clinical practice [31,32,33]. Randomized controlled trials (RCTs) inclusion criteria frequently exclude the enrolment of essential parts of the patient population. The comparators in RCTs do not reveal standard available care [32,34,35,36]. RCTs are the gold standard in efficacy and safety occupying a short period, occurring under controlled conditions, and using selected populations under idealized conditions [31,32,34,37]. RCTs can also be prone to systematic errors, leading to biased estimates when generalizing to real-world settings, and this is especially problematic in many public health interventions [38].

The present study aims to investigate if the application of one-year adjuvant trastuzumab, in cases of early HER2-positive breast cancer, is cost-effective, using real-world data. The objectives of the study in a real-world setting are (a) the cost-effectiveness analysis of the use of trastuzumab (all trastuzumab arms), (b) the cost-effectiveness analysis of the use of treatment regimens not including trastuzumab, (c) to examine the three trastuzumab arms in order to investigate the ICER of each, and (d) to compare the entire sample with trastuzumab with the three trastuzumab arms. The significance of the study is fourfold. Firstly, the current analysis is one of the first cost-effectiveness analyses, using real-world clinical input data, of adjuvant trastuzumab for one year in early female HER2-positive breast cancer and the first one in a Cyprus setting. Secondly, this study is just the second one in Europe and the fourth one worldwide ever carried out using real-world data. Moreover, an economic evaluation is applied to the use of trastuzumab (real-world) in an adjuvant setting. In addition, it examines three different treatment schemes to identify the best practice therapy. It is the first time that research in a real-world setting has examined three various treatment schemes to investigate the cost-effectiveness of each. It is also the first time that the entire sample with trastuzumab is being compared to the three treatment regimens.

## 2. Materials and Methods

### 2.1. Methodology, Subjects and Treatments

Using a Markov model [39], a cost-effectiveness analysis [35] was carried out over 20 years and for a lifetime horizon. The scenarios of one-year adjuvant therapy with trastuzumab in early female HER2-positive breast cancer and standard adjuvant chemotherapy (e.g., docetaxel, doxorubicin, cyclophosphamide combination) were examined. A simple randomized sampling of 900 women with early breast cancer was included in the study. In total, 148 females with early HER2-positive breast cancers, who received one year of adjuvant trastuzumab treatment, with starting dates from 2005 to 2012, recovered. In addition, 30 patients with early HER2-positive breast cancer, who received standard adjuvant chemotherapy (e.g., docetaxel, doxorubicin, cyclophosphamide) before 2005, recovered. The 30 patients were the control group. Before 2005, only a few women with early breast cancer received HER2 screening. This was why a relatively small control group was used. Patients treated with trastuzumab were separated (total *N* = 148) into one of the following three schemes: (i) anthracyclines and then trastuzumab (*N* = 38), (ii) no anthracyclines and then trastuzumab (*N* = 14), and (iii) anthracyclines, taxanes, and trastuzumab *(N* = 96). Each group was compared to the control group (*N* = 30). The same cost-effectiveness analysis was carried out, as above, over 20 years and for a lifetime horizon.

**Figure 1:**
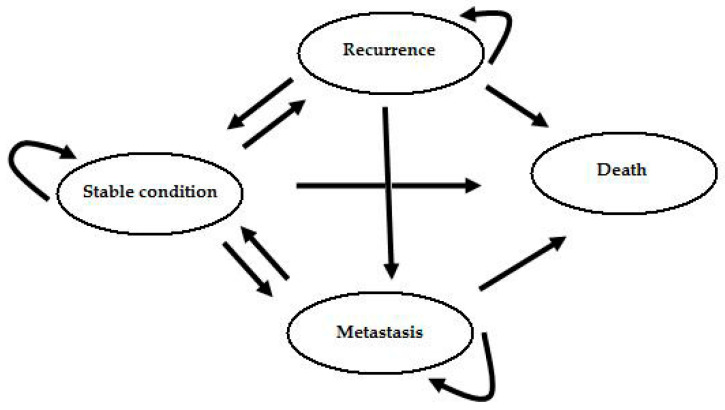
Markov model—health state structure.

The four health states used in the Markov model were stable condition, recurrence, metastasis, and death (Figure 1). Data on the occurrence of relapses, metastasis, and death were recorded (Table 1). In addition, the chances of the transition of patients from one state to another were calculated. Table 2 shows the baseline parameters of the cost-effectiveness analysis.

### 2.2. Cost Calculations

The cost was estimated using a hypothetical third-party payer on behalf of the Cyprus Health System. Such analysis includes only the direct costs associated with the treatment and follow-up of patients [42]. Costs were calculated based on costs in the Cyprus Health System. The prices of the medicines used were the retail prices based on the Single Price List as of 2 October 2017 [43].

The trastuzumab-related costs included costs of diagnostic tests, blood tests, the cost to determine HER2 status (immunohistochemistry (IHC) tests, FISH tests), medications and supportive medications, hospital cost when receiving the therapy, and cardiac monitoring costs.

### 2.3. Data Collection

The survey was based on data collected by the Bank of Cyprus Oncology Centre. A code identified each patient in the study. The research is independent and has neither been funded nor has any relationship with any pharmaceutical company. The use of trastuzumab for the treatment of adult patients with early HER2-positive breast cancer was undertaken by the current treatment protocols of the Cyprus Ministry of Health.

### 2.4. Statistical Analysis for Cost-Effectiveness Analysis

Mean and standard deviation (SD) were used to describe the quantitative variables. Using the Kolmogorov–Smirnov criterion, the distributions of the quantitative variables were checked and found to be normally distributed. Pearson’s correlation coefficient (r) was used to test the relationship between two quantitative variables. The correlation is considered low when the correlation coefficient (r) ranges from 0.1 to 0.3, moderate when the correlation coefficient ranges from 0.31 to 0.5, and high when the coefficient is greater than 0.5. Student’s *t*-test was used to compare quantitative variables between the two groups. We use a parametric analysis of variance (ANOVA) for the comparison of the means of groups. For the control of type I error, Bonferroni correction was used for multiple comparisons, according to which the significance level is 0.05/k (k = number of comparisons). Linear regression analysis by the sequential integration/removal process (stepwise) was used to find independent factors related to the cost/QALY ratio, which resulted in dependency factors (β) and their standard errors (SE). To compare proportions, Fisher’s exact test was used where necessary.

Significance levels were two-sided and statistical significance was set at 0.05. SPSS 22.0 was used for statistical analysis [44]. Using the TreeAge Pro 2017 program [45], Markov models were designed, with a cycle of 1 year, to assess long-term outcomes, quality of life, and the cost of treatment regimens with and without Trastuzumab. The time horizon of the study was 20 years or the lifetime of the patients. Transition probabilities and costs were calculated based on this sample. The QALYs used were found in the literature [40,41]. QALYs were obtained from the EQ-5D tool. EQ-5D includes five domains: mobility, self-care, usual activities, pain/discomfort, and anxiety/depression. Each domain is divided into three severity levels, corresponding to no problems, some problems, and extreme problems. It is possible to obtain social values for each, thereby generating a score of health state valuations, with the highest value of 1. This score was used as QALY in our analysis [46]. Both the costs and the QALYs were discounted at a fixed discount rate of 3%. A half-cycle correction was used in the analyses. The results are presented with the incremental cost-effectiveness ratio (ICER), at a cost per QALY earned. Both one-way deterministic and multiway probabilistic sensitivity analyses were performed to check the uncertainty of the parameters. Simple sensitivity analyses were based on ±10% variation in the values of the main variables of the baseline scenario. The probabilistic sensitivity analysis was performed with 1000 Monte Carlo iterations and with hypothetical acceptance thresholds of €20,000, €40,000, and €60,000/QALY gained. According to the references from the literature [47], random values were obtained from beta distributions for QALYs and from gamma distributions for variable costs.

According to the report, interventions costing less than three times the value of GDP per capita for each DALY (disability-adjusted life year) avoided are cost-effective [48,49,50,51]. Cyprus’ real GDP per capita in 2017 was €23,700 [52].

### 2.5. Ethics Approval

The study was approved by the Ethics Committee (ΕΕΒΚ ΕΠ 2017.01.04.01), the Office of the Commissioner for Personal Data Protection (3.28.75), and the Ministry of Health (5.34.01.7.4E) of the Republic of Cyprus. The General Director of the Bank of Cyprus Oncology Centre granted permission to conduct the study (ΠΕ/AΓ/5992).

## 3. Results

Females with early HER2-positive breast cancers, who received one year of adjuvant trastuzumab treatment, had a mean age of 52.3 years (SD = 10.2 years) at the initiation of treatment. More specifically, 83.1% of the patients had received trastuzumab. Additionally, 71.9% of patients had had hormone therapy, and 82.6% had undergone radiotherapy. The mean follow-up period was 8.0 years (SD = 2.7), with a median equal to 7.5 years (interquartile range from 6.1 to 8.9 years). During the follow-up period, recurrence (local and systemic) occurred in 17 (11.5%) patients and two patients died (1.4%). Among the 148 patients, and if we isolate the more important systemic relapses, only 8 patients had systemic relapses; therefore, a percentage of 5.41%. The mean time interval between patients’ diagnosis and recurrence was 4.3 years (SD = 2.6 years) with a median equal to 4.1 years (interquartile range from 2.3 to 6.4 years). Sample demographic and clinical characteristics of the four treatment groups are presented in Table 3.

### 3.1. The Costs of Examinations and Treatment

Table 4 shows the costs of treatment and tests for the control arm and trastuzumab arms: (a) all trastuzumab arms, and b) the three subgroups of trastuzumab arms.

The mean cost of the examinations and the 1st chemotherapy was €2786.90 (SD = €604.85) for the control arm, and €2632.91 (SD = €905.46) for all trastuzumab arms (N = 148). The mean cost of the 2nd chemotherapy was €5105.47 (SD = €238.88) for the control arm, while the mean cost of all trastuzumab arms was €38,069.95 (SD = €6226.03).

After all trastuzumab arms (N = 148) were separated into three subgroups of trastuzumab arms, the analysis showed that the mean cost of the tests and the 1st chemotherapy was €3097.47 (SD = €396.03) for the subgroup anthracyclines and then trastuzumab, €182.14 (SD = €63.87) for the subgroup no anthracyclines and trastuzumab, and €2806.43 (SD = €452.88) for the subgroup anthracyclines, taxanes, and trastuzumab.

The mean cost of trastuzumab treatments (three subgroups) was €38,210.71 (SD = €5499.80) for the subgroup anthracyclines and then trastuzumab, €37,240.37 (SD = €6380.80) for the subgroup no anthracyclines and trastuzumab, and €38,135.20 (SD = €6520.39) for the subgroup anthracyclines, taxanes, and trastuzumab.

### 3.2. The Results of the Cost-Effectiveness Analysis over 20 Years and for a Lifetime Horizon

After the initial estimation of cost-effectiveness for all trastuzumab arms and the control arm, the trastuzumab arm was divided into three comparative subgroups. Two predictive scenarios were applied to a time scale of 20 years and also to a lifetime on trastuzumab (Table 5).

In the 20-year scenario, the cost-effectiveness analysis based on all trastuzumab arms showed that trastuzumab-free treatment resulted in 1.70 QALYs at a total cost of €3978.18. In comparison, trastuzumab treatment resulted in 2.72 QALYs at a total cost of €45,420.45. Compared to trastuzumab-free treatment, trastuzumab treatment (all trastuzumab arms) had an ICER of €40,436.10/QALY gained. On the other hand, the cost-effectiveness analysis based on the three subgroups of trastuzumab arms showed that treatment without trastuzumab yielded 1.42 QALYs at a total cost of €3978.18, treatment with anthracyclines and then trastuzumab yielded 3.46 QALYs at a total cost €41,308.19, treatment with no anthracyclines and trastuzumab produced 3.14 QALYs at a total cost of €43,664.83, and treatment with anthracyclines, taxanes, and trastuzumab resulted in 3.21 QALYs at a total cost €47,304.25. In comparison to treatment without trastuzumab, treatment with anthracyclines and then trastuzumab had an ICER of €18,301.55 per gained QALY, treatment with no anthracyclines and trastuzumab had an ICER of €23,138.90/gained QALYS, and treatment with anthracyclines, taxanes, and trastuzumab had an ICER of €24,254.73 per gained QALY. From the three subgroups of trastuzumab arms, the most cost-effective was that of anthracyclines and then trastuzumab, as it was lower in cost and yielded more QALYs.

In the lifetime horizon scenario, the cost-effectiveness analysis based on all trastuzumab arms showed that trastuzumab-free treatment resulted in 1.82 QALYs at a total cost of €3978.18. In comparison, trastuzumab treatment resulted in 4.15 QALYs at a total cost of €45,420.45. Compared to trastuzumab-free treatment, trastuzumab treatment had an ICER of €17,753.85/QALY gained. On the other hand, the cost-effectiveness analysis based on the three subgroups of trastuzumab arms showed that trastuzumab-free treatment resulted in 1.51 QALYs at a total cost of €3978.18, anthracyclines and then trastuzumab treatment resulted in 5.68 QALYs at a total cost of €41,308.19, treatment with no anthracyclines and trastuzumab resulted in 4.46 QALYs at a total cost of €43,664.83, and treatment with anthracyclines, taxanes, and trastuzumab resulted in 4.82 QALYs total cost €47,304.25. In comparison with treatment without trastuzumab, the treatment with anthracyclines and then trastuzumab had an ICER of €8954.97 per gained QALY, treatment with no anthracyclines and trastuzumab had an ICER of €13,445.63/gained QALY, and treatment with anthracyclines, taxanes, and trastuzumab had an ICER of €13,111.44 per gained QALY. Of the three subgroups of trastuzumab arms, the most cost-effective was that of anthracyclines and then trastuzumab, as it was lower in cost and yielded more QALYs.

### 3.3. Sensitivity Analysis Based on All Trastuzumab Arms

Initially, one-way sensitivity analyses were performed (for both 20-year and lifetime horizons), varying some of the parameters of the baseline scenario by +10% or −10% of the initial value. These results are given in Table A1 (Appendix A).

For both 20-year and lifetime horizons, the cost-effectiveness analysis was more sensitive to changes in the total cost of treatment with trastuzumab and the utility weight of stable disease. However, the changes they caused in the ICERs were to the extent of ±10%.

For the 20-year horizon, after a probabilistic sensitivity analysis, it was found that the cost of treatment with trastuzumab ranged from €25,497.16 to €68,099.47 and the QALYs from 0.42 to 4.26. Correspondingly, the cost of treatment without trastuzumab ranged from €537.58 to €13,631.63 and the QALYs from 0.29 to 2.63. Using a hypothetical acceptance threshold of €20,000/QALY gained, 99.0% of the simulations showed treatment without trastuzumab to be more cost-effective. In contrast, with a hypothetical acceptance threshold of €40,000/QALY gained, 49.1% of the simulations found trastuzumab to be more cost-effective, with a hypothetical acceptance threshold of €60,000/QALY gained, 82.6% of the simulations found trastuzumab treatment more cost-effective.

For the lifetime horizon, after a probabilistic sensitivity analysis, it was found that the cost of treatment with trastuzumab ranged from €25,952.31 to €74,110.58 and the QALYs from 0.76 to 6.55. Correspondingly, the cost of treatment without trastuzumab ranged from €419.93 to €11,539.53 and the QALYs from 0.37 to 2.83. Using a hypothetical acceptance threshold of €20,000/QALY gained, 66.6% of the simulations showed trastuzumab treatment to be more cost-effective. Similarly, with a hypothetical acceptance threshold of €40,000/QALY gained, 96.6% of the simulations found trastuzumab treatment more cost-effective, and with a hypothetical acceptance threshold of €60,000/QALY gained, 99% of the simulations found trastuzumab treatment more cost-effective.

### 3.4. Sensitivity Analysis Based on the three Subgroups of Trastuzumab Arms

Initially, one-way sensitivity analyses were performed (for both 20-year and lifetime horizons), varying some of the baseline parameters by + 10% or −10% of the initial value. These results are presented in Table A2 and Table A3 (Appendix A).

For both the 20-year and lifetime horizons, the cost-effectiveness analysis was more sensitive to changes in the cost of each trastuzumab treatment and the utility of stable disease.

For the 20-year horizon, after a probabilistic sensitivity analysis, it was found that the cost of treatment with anthracyclines and after trastuzumab ranged from €24,659.89 to €61,565.49 and QALYs from 0.60 to 5.33. The cost of treatment with no anthracyclines and trastuzumab ranged from €25,623.21 to €71,972.16, and QALYs from 0.64 to 4.77, and the cost of treatment with anthracyclines, taxanes, and trastuzumab ranged from €26,001.80 to €68,946.07 and QALYs from 0.48 to 4.98. Accordingly, treatment costs without trastuzumab ranged from €679.13 to €13,331.85 and QALYs from 0.25 to 2.19. With a hypothetical acceptance threshold of €20,000/QALY gained, 55.8% of the simulations found treatment with anthracyclines followed by trastuzumab more cost-effective, 34.7% treatment without trastuzumab, 5.5% treatment with no anthracyclines and trastuzumab, and the remaining 4% treated with anthracyclines, taxanes, and trastuzumab. In contrast, with a hypothetical acceptance threshold of €40,000€/QALY gained, 89.1% of the simulations showed treatment with anthracyclines followed by trastuzumab to be more cost-effective, 2.3% treatment with trastuzumab, 5.8% treatment with no anthracyclines and trastuzumab, and the remaining 2.8% treatment with anthracyclines, taxanes, and trastuzumab. Using a hypothetical acceptance threshold of €60,000/QALY gained, 95.1% of the simulations found treatment with anthracyclines followed by trastuzumab to be more cost-effective, 0.3% with no trastuzumab treatment, 3.4% with no anthracyclines and trastuzumab, and the remaining 1.2% treatment with anthracyclines, taxanes, and trastuzumab (Table 6).

For the lifetime horizon, after a probabilistic sensitivity analysis, it was found that the cost of treatment with anthracyclines and after trastuzumab ranged from €25,759.28 to €65,416.49 and QALYs from 0.87 to 8.95. The cost of treatment with no anthracyclines and trastuzumab ranged from €27,124.29 to €70,008.66 and QALYs from 0.92 to 6.76, while the cost of treatment with anthracyclines, taxanes, and trastuzumab ranged from €27,099.57 to €74,217.63 and QALYs from 0.63 to 7.65. Respectively, the cost of treatment without trastuzumab ranged from €740.62 to €12,362.05 and the QALYs from 0.23 to 2.38. With a hypothetical acceptance threshold of €20,000/QALY gained, 95.5% of the simulations found treatment with anthracyclines and then trastuzumab more cost-effective, 3.2% trastuzumab-free treatment, 1% treatment with no anthracyclines and trastuzumab, and the remaining 0.3% treatment with anthracyclines, taxanes, and trastuzumab. Using a hypothetical acceptance threshold of €40,000/QALY gained, 98.7% of the simulations found therapy with anthracyclines and then trastuzumab more cost-effective, 0.3% trastuzumab-free treatment, 0.9% with no anthracyclines and trastuzumab, and the remaining 0.1% anthracyclines, taxanes, and trastuzumab treatment. With a hypothetical acceptance threshold of €60,000/QALY gained, 99% of the simulations yielded therapy with anthracyclines and then trastuzumab more cost-effective, 0% trastuzumab-free treatment, 1% treatment with no anthracyclines and trastuzumab, and 0% treatment with anthracyclines, taxanes, and trastuzumab (Table 6).

### 3.5. Impact of the Time Horizon on the ICER of Treatment

**Figure 2:**
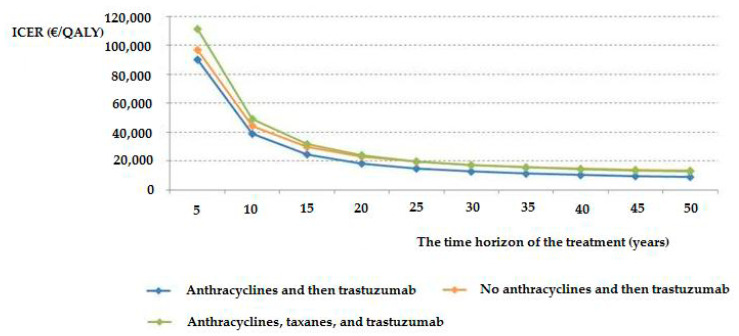
The ICERs of the three different trastuzumab subgroups for various time horizons of the analysis.

The ICERs of all trastuzumab arms and the three different trastuzumab subgroups were decreased when the time horizon of the analysis was extended from 5 to 10 years and from 10 to 15 years. After 15 years, the decline in ICERs (i.e., increasing the cost-effectiveness of trastuzumab therapy) continues, although at a slower rate than the initial rate (Figure 2).

### 3.6. Multivariate Analysis with the Dependent Variable of the Cost/QALY Ratio

For each patient, a calculation was made of the ratio of cost/QALY, which was used as a dependent variable in a model of multiple linear regression to find which factors were influential to a significant degree. The independent variables used were age at the time of diagnosis, somatometric data of patients, data related to their disease, and data related to their treatment. The following results were obtained using the sequential integration-removal method (stepwise method; Appendix B Table A4).

Based on all trastuzumab arms (N = 148), it was found that the body surface area (BSA), the degree of positive expression of progesterone receptors, radiotherapy, and the number of infiltrated lymph nodes were independently related to the cost/QALY ratio. Specifically, as patients’ BSA increased, so did the cost/QALY ratio (*p* < 0.001). Additionally, the higher the positive expression of progesterone receptors, the lower the cost/QALY ratio (*p* = 0.002). Furthermore, patients who underwent radiotherapy had a 14,604-unit higher cost/QALY ratio compared to patients who had not received radiotherapy (*p* = 0.006). Moreover, as the number of infiltrated lymph nodes (based on Tumor-Node-Metastasis (TNM) classification) in patients increased, so did the cost/QALY ratio (*p* = 0.025).

On the other hand, based on the three subgroups, for the patients who received anthracyclines and then trastuzumab, the age at diagnosis and BMI were found to be related independently to the cost/QALY ratio. Correctly, it was shown that the older the patients were at diagnosis, the lower the cost/QALY ratio (*p* = 0.006), while the higher the BMI of patients was, the higher the cost/QALY ratio (*p* = 0.012).

For the patients who received no anthracyclines and then trastuzumab, only radiotherapy was found to be independently related to the cost/QALY ratio. In particular, patients who had undergone radiotherapy had a higher cost/QALY ratio of 35,142.76 points than patients who had not received radiotherapy (*p* = 0.006).

For the patients who received anthracyclines, taxanes, and trastuzumab, the BSA, the degree of positive expression of progesterone receptors, radiotherapy, and the number of filtered lymph nodes were found to be independently related to the cost/QALY ratio. Specifically, it was found that the higher the BSA of patients, the higher the cost/QALY ratio (*p* < 0.001). Additionally, it was shown that the higher the degree of positive expression of progesterone receptors, the lower the cost/QALY ratio (*p* = 0.007). Furthermore, it was found that patients who had received radiotherapy had a higher cost/QALY ratio of 20,007.28 than patients who had not undergone radiotherapy (*p* = 0.012). Moreover, as the number of filtered lymph nodes (based on TNM) increased, the cost/QALY ratio increased (*p* = 0.004).

### 3.7. Correlation of the Cost/QALY Ratio with the Weight and Age of Patients at Diagnosis

Age at diagnosis was not found to be significantly related to the cost/QALY ratio. In contrast, there was a significant positive correlation between weight and cost/QALY ratio across the whole patient sample (*N* = 178): both those who had taken trastuzumab (N = 148; *p* < 0.001) and patients independent of the three different trastuzumab subgroups (*p* < 0.001). Therefore, higher weight values were associated with a higher cost/QALY ratio.

### 3.8. Correlation of the Cost/QALY Ratio with the Stage and Grade of Patients

The cost/QALY ratios were not significantly different, depending on the grade and stage of patients, neither across the whole sample (*N* = 178) nor in those treated with trastuzumab (*N* = 148) or those who were not (*N* = 30). After all trastuzumab arms (*N* = 148) were separated into three subgroups of trastuzumab arms, the correlation showed that in patients who took no anthracyclines and then trastuzumab, the cost/QALY ratio (€60,829.17/QALY, SD: €6894.37) was significantly lower in those with Grade 3 (*p* = 0.010) than in those with Grades 1–2. Additionally, the cost/QALY ratio differed significantly in patients who took anthracyclines, taxanes, and trastuzumab, depending on the stage of their disease (*p* = 0.004). Specifically, after Bonferroni correction, it was found that patients in Stage I had a significantly higher cost/QALY ratio (€80,886.69/QALY, SD: €12,382.52) than patients in Stage III (€70,421.39/QALY, SD: €10,627.24) (*p* = 0.004).

### 3.9. Correlation of the Cost/QALY Ratio with Age at Treatment Initiation

The cost/QALY ratios were not significantly different, depending on age at the start of treatment, neither across the total sample (*N* = 178) nor in those treated with trastuzumab (*N* = 148) or not (*N* = 30), after all trastuzumab arms (*N* = 148) were separated into three subgroups of trastuzumab arms.

## 4. Discussion

The study aims to investigate, using real-world data, if the application of one-year adjuvant trastuzumab, in cases of early HER2-positive breast cancer, is beneficial to the health outcomes of women concerning the costs (cost-effectiveness analysis). This study is one of the few cost-effectiveness analyses published worldwide that is based upon real-world data. Regarding the first and second objectives of the study, the findings suggest that for the 20-year horizon, with only a hypothetical acceptance threshold of €60,000/QALY gained, trastuzumab treatment (all trastuzumab arms) was found to be more cost-effective (ICER of €40,436.10/QALY). For the lifetime horizon, using hypothetical acceptance thresholds of €20,000/QALY gained, €40,000/QALY gained, and €60,000/QALY gained, trastuzumab treatment (all trastuzumab arms) was found to be more cost-effective (ICER of €17,753.85/QALY gained). Based on all trastuzumab arms, it was found that body surface area (BSA), the degree of positive expression of progesterone receptors, radiotherapy, and the number of infiltrated lymph nodes (based on Tumor-Node-Metastasis (TNM) classification) were independently related to the cost/QALY ratio. Specifically, as patients’ BSA increased, so did the cost/QALY ratio. Additionally, the higher the positive expression of progesterone receptors, the lower the cost/QALY ratio. Furthermore, patients who underwent radiotherapy had a 14,604-unit higher cost/QALY ratio compared to patients who had not received radiotherapy. Moreover, as the number of infiltrated lymph nodes (TNM) of patients increased, so did the cost/QALY ratio. With respect to the third objective of the study, patients treated with trastuzumab in this study were assigned one of the following three regimens (i) anthracyclines and then trastuzumab, (ii) no anthracyclines and then trastuzumab, and (iii) anthracyclines, taxanes, and trastuzumab. This study indicates that for both the 20-year and the lifetime horizons, with hypothetical acceptance thresholds of €20,000/QALY gained, €40,000/QALY gained, and €60,000/QALY gained, the most cost-effective of the three subgroups of trastuzumab arms was that of anthracyclines and then trastuzumab (an ICER of €18,301.55 per gained QALY and €8954.97 per gained QALY, respectively). For patients who received anthracyclines and then trastuzumab, their age at diagnosis and BMI were found to be independently related to the cost/QALY ratio. Specifically, it was shown that the older the patients were at the time of diagnosis, the lower the cost/QALY ratio, and the higher the BMI of patients, the higher the cost/QALY ratio. As concerns the last objective, the ICERs of all trastuzumab arms and the three different trastuzumab subgroups decreased when the time horizon of the analysis was extended from 5 to 10 years and 10 to 15 years. After 15 years, the decline in ICERs (i.e., increasing the cost-effectiveness of trastuzumab therapy) continued, although at a slower rate than the initial rate. There was a significant positive correlation between weight and cost/QALY ratio in the entire patient sample, those who had taken trastuzumab, and patients independent of the three different trastuzumab subgroups. Therefore, higher weight values were associated with a higher cost/QALY ratio.

The vast majority of previous studies were based on the assumption that data collected from clinical trials are equal to those from a real-world setting. Randomized controlled trials have internal validity but usually have limited external validity. A real-world environment with a simple random sampling (unselected sample and unselected treatment scheme) differs from a trial setting (selected sample and treatment schemes), and, as a consequence, it is likely to see different results, which are essential for reimbursement decisions [32]. Even more, clinical trials have stringent inclusion criteria, which make it difficult to extrapolate data from the general populations seen in clinical practice [53]. There was a difference between studies in ICER. There are many reasons, and these vary by study. Different drug costs, cost-effectiveness modeling, study design, country-related differences in health care systems, underlying assumptions, and the origin of the data: if they were from a real-world setting or clinical trials [29,54]. The difference in these results may be due to the different resources used: for example, the probability of disease progression [55].

Another real-world cost-effectiveness study conducted in Canada (2012) used a Markov model. The study concluded that there was a gain of 1.38 QALY or 1.17 LYG at the cost of CA$18,133 per patient. The ICER (QALY) was CA$13,095/QALY, while ICER (LYG) was CA$15,492/LYG. The research concluded that, in the long run, it is a cost-effective treatment [19]. If we convert Canadian dollars to euro (1€ = CA$1.4320) [56], the ICER would be €9144.55/QALY. This result is less than in our study. The difference was in the time horizon. The time horizon for Hedden et al. [19] was 28 years versus the 20-year and lifetime horizons of our study. In other real-world research in Taiwan (2016) [21], a Markov model was used over a time horizon of 20 years. The model showed that treatment with trastuzumab yielded 1631 QALY compared to treatment without trastuzumab. ICER was US$51,863/QALY gained in the baseline scenario. Monte Carlo simulation of all variables simultaneously showed that cost-effectiveness at the USD67,065 “willingness-to-pay” threshold was 50% for one year of adjuvant trastuzumab. The study concluded that trastuzumab was a cost-effective, one-year adjuvant treatment for early HER2-positive breast cancer [21]. If we convert USD to euros (€1 = US$1.0801) [56], the ICER is about €48,016.85/QALY. This result is quite similar to that of our study, which was €40,436.10/QALY for a 20-year time horizon. The difference in our study was that for the 20-year horizon, after a probabilistic sensitivity analysis, it was found that, with a hypothetical acceptance threshold of €60,000/QALY gained, 82.6% of the simulations found trastuzumab treatment more cost-effective. Another real-world setting study was conducted in the Netherlands [26]. A Markov model with a lifetime horizon was used with three scenarios: (a) real-world scenario, (b) trial scenario, and (c) guideline scenario. The corresponding ICERs were €4304/QALY, €6382/QALY, and dominance, respectively. The study concluded that adjuvant trastuzumab could, in real terms, be considered cost-effective for all three scenarios. In our research, the ICER was four times higher than in Seferina’s study [26]. After a probabilistic sensitivity analysis, it was found that using a hypothetical acceptance threshold of €60,000/QALY gained, 99% of the simulations found trastuzumab treatment more cost-effective. On the other hand, Seferina’s study indicated that using a threshold of €80,000 per QALY gained, the probability that one-year adjuvant trastuzumab was cost-effective was 99% or higher in all three scenarios. Previous studies using a 20-year horizon and clinical trial data showed that one-year adjuvant trastuzumab is a cost-effective regimen in several countries. In the United States (2007), a cost-effectiveness analysis estimated an ICER of US$34,201/QALY (€31,664.66/QALY) [14,56]. In Norway, an analysis by Norum et al. found that the ICER was €19,176/QALY (20% OS improvement) and €44,934/QALY (10% OS improvement) [25]. Three previous studies using lifetime-horizons and clinical trial data showed that one-year adjuvant trastuzumab was a cost-effective regimen [11,22,24]. In Australia, Millar and Millward (2007) projected the cost-effectiveness of trastuzumab to be AUD22,793/QALY (€13,929.60/QALY) [24,56]. Chen and colleagues (2009, China) reported that the ICER was USD7676/QALY (€7106.75/QALY), USD8,049/QALY (€7452.09/QALY) and USD8046/QALY (€7449.31/QALY), in Beijing, Shanghai, and Guangzhou, respectively [11,56]. Leung and colleagues (2016, New Zealand) projected the ICER to be below NZD45,000 (€26,309.64) (ER-negative/PR-negative) and up to nearly NZD100,000 (€58,465.86) (ER-positive/PR-positive) per QALY. Patients with positive ER and positive lymph nodes did not show a favorable cost-effectiveness ratio. On the contrary, cases with negative ER/PR subtypes, up to the age of 69 years, showed a favorable cost-effectiveness ratio [22,56]. Liberato and colleagues showed that one-year adjuvant trastuzumab was a cost-effective regimen. Using clinical-trial data, they estimated the cost per quality-adjusted life-year gained over a 15-year horizon to be €14,861/QALY from the perspective of the Italian health care system and USD18,970/QALY (€17,563.19/QALY) from a US healthcare perspective [23,56]. In contrast, another three studies using lifetime horizons and clinical-trial data showed that one-year adjuvant trastuzumab is not a cost-effective regimen [10,15,16]. In Colombia, Buendia and colleagues (2013) projected the ICER to be USD71,491/QALY (€66,189.24/QALY) [10,56]. In the Philippines, Genuino and colleagues (2019) reported that the ICER was PHP453,505/QALY(€8252.30/QALY) [15,56]. In another recently published analysis based on Markov models, Gershon and colleagues (2019, subSaharan Africa) projected that the ICER ranged from USD19,534/QALY (€18,085.36/QALY) in South Africa to USD 21,697/QALY (€20,087.95/QALY) in Nigeria [16,56]. One Iranian study (2014), using a 20-year horizon and clinical-trial data, showed that one-year adjuvant trastuzumab was not a cost-effective regimen, with the ICER being USD51,302/QALY (€47,497.45/QALY) [9,56]. In a recently-published study, Hajjar and colleagues (2019, USA) used Markov models to simulate four adjuvant therapy options, with a lifetime time horizon and using clinical-trial data, and showed that one-year adjuvant paclitaxel and trastuzumab was a cost-effective regimen. The four adjuvant therapy options were (1) adjuvant paclitaxel and trastuzumab (TH), (2) doxorubicin, cyclophosphamide, paclitaxel, and trastuzumab (ACTH), (3) docetaxel, carboplatin, and trastuzumab (TCH), and (4) no adjuvant trastuzumab (NT). The study yielded an ICER of USD10,584/QALY (approximately €9799.09/QALY; ages 40–49) and USD84,981/QALY (approximately €78,678.83/QALY; ages 80+) [17,56]. The adjuvant paclitaxel and trastuzumab treatment were included in our study, in the no-anthracyclines and then trastuzumab arm.

### Strengths and Limitations

A significant strength of this study is that patients treated with trastuzumab (all trastuzumab arms) were separated in one of the following three schemes: (i) anthracyclines and then trastuzumab, (ii) no anthracyclines and then trastuzumab, and (iii) anthracyclines, taxanes, and trastuzumab. A cost-effectiveness analysis was carried out for each of the three distinct groups and for the whole sample. Moreover, another significant strength of this study is the use of three different thresholds (€20,000, €40,000, and €60,000) and two different time horizons (20 years and lifetime horizon). No other published study has involved all these correlations. There were several limitations to our analysis. In our studies, we used utilities from other countries because, in Cyprus, there were no health-related quality-of-life data for breast cancer patients. Second, we used direct costs rather than societal costs, which would include costs such as caregiver time and lost productivity. Using direct costs was assumed to be most relevant to healthcare payers, considering coverage decisions, and was felt to be more comparable with previously published studies. Third, before 2005, only a few women with early breast cancer received HER2 screening. This was why a relatively small sample was used as the control group. Fourth, our analysis was based on estimates of effectiveness in real-world settings rather than in controlled trials. It was likely that the observed effectiveness of adjuvant treatment in real-world settings would be less favorable than those observed in clinical trials. Fifth, correlation does not imply causality and, therefore, the results should interpret with some caution. We could only talk about causality in the event of a randomized clinical trial. Finally, since there was no acceptable threshold in Cyprus, we used three thresholds based on WHO recommendations [48]. The rapidly increasing number of cancer drugs and different treatment arms is one of the major factors in the problems of the economic sustainability of cancer treatments in various healthcare systems [57]. In several European countries, there are fundamental price differences in comparison to the actual cost of cancer drugs [58,59]. Performance-based risk-sharing arrangements for the introduction and continuation of the use of a cancer drug in a healthcare system, based on real-world effectiveness and real-world cost-effectiveness evaluations, could be used to mitigate the hazards of financial sustainability and inequality in the access to cancer care in different countries [60]. The present study can be improved upon in the future. First, it is recommended to repeat the modeling using local breast cancer health utilities and more long-term survival data, once they become available. Cardiovascular risk factors can further investigate heterogeneity. Moreover, direct nonmedical costs can be included. Finally, a microcosting study of actual economic data can yield a more accurate estimation [61].

## 5. Conclusions

It is the first time that there has been a real-world examination of three different treatment subgroups and a comparison of the entire sample with them. In conclusion, adjuvant trastuzumab for one year in early female HER2-positive breast cancer in a real-world setting can be considered cost-effective. The most cost-effective of the three subgroups (anthracyclines and then trastuzumab, no anthracyclines and then trastuzumab, and anthracyclines, taxanes, and trastuzumab) was that of anthracyclines and then trastuzumab.

## Figures and Tables

**Table 1:** Transition probabilities (TP) used in the model.

Health States		All Trastuzumab Arms		Control Group		Three Subgroups of Trastuzumab Arms					
Transition		With Trastuzumab		Without Trastuzumab		Anthracyclines and then Trastuzumab		No Anthracyclines and Trastuzumab		Anthracyclines, Taxanes, and Trastuzumab	
from:	to:	*N*	TP	*N*	TP	*N*	TP	*N*	TP	*N*	TP
Stable	Stable	400	0.946	58	0.744	98	0.925	37	0.949	265	0.953
Stable	Recurrence	6	0.014	3	0.038	4	0.038	1	0.026	1	0.004
Stable	Metastasis	16	0.038	9	0.115	4	0.038	1	0.026	11	0.040
Stable	Death	1	0.002	8	0.103	0	0.000	0	0.000	1	0.004
Recurrence	Stable	5	0.714	3	1.000	3	0.750	1	1.000	1	0.500
Recurrence	Recurrence	1	0.143	0	0.000	0	0.000	0	0.000	1	0.500
Recurrence	Metastasis	1	0.143	0	0.000	1	0.250	0	0.000	0	0.000
Recurrence	Death	0	0.000	0	0.000	0	0.000	0	0.000	0	0.000
Metastasis	Stable	8	0.571	9	1.000	3	0.750	0	0.000	5	0.625
Metastasis	Metastasis	5	0.357	0	0.000	1	0.250	2	1.000	2	0.250
Metastasis	Death	1	0.072	0	0.000	0	0.000	0	0.000	1	0.125

*N*: Number of transitions; TP: Transition probabilities.

**Table 2:** The parameters of the basic scenario.

Parameters	Value of Basic Scenario					
	Control Group	All Trastuzumab Arms	Three Subgroups of Trastuzumab Arms			Reference
	without Trastuzumab	with Trastuzumab	Anthracyclines and Then Trastuzumab	No Anthracyclines and Trastuzumab	Anthracyclines, Taxanes, and Trastuzumab	
Cost						
Total costs *	€ 3978.18	€ 45,420.45	€ 41,308.19	€ 43,664.83	€ 47,304.25	‡
Utility weights						
EQ-5D For stable condition	0.620					a
EQ-5D For recurrence	0.767					a
EQ-5D For metastasis	0.692					a
Deducted for stable condition/recurrence	–0.215					c
Deducted for metastasis	–0.0519					c
Discount rate						
Cost	3%					b
QALY	3%					b

* The trastuzumab-related costs included costs of diagnostic tests, blood tests, the cost to determine HER2 status (immunohistochemistry (IHC) tests, FISH tests), medications, and supportive medications, hospital cost when receiving the therapy, and cardiac monitoring costs. ‡ Primary data from the present study, a from ref. [40], b from ref. [35], c from ref. [41].

**Table 3:** Sample demographic and clinical characteristics.

Sample Characteristics		Treatment			
		No Trastuzumab	Anthracyclines and Then Trastuzumab	No Anthracyclines and Then Trastuzumab	Anthracyclines, Taxanes, and Trastuzumab
Number of patients		30	38	14	96
Height, cm (SD)		158.0 (9.6)	157.6 (8.4)	158.7 (8.1)	161.0 (9.5)
Weight, kg (SD)		68.9 (14.4)	69.9 (10.4)	68.1 (12.1)	69.8 (12.4)
BSA, m^2^ (SD)		1.70 (0.17)	1.71 (0.11)	1.70 (0.15)	1.73 (0.15)
BMI, mean (SD)		27.89 (7.08)	28.47 (5.99)	27.16 (5.36)	27.16 (5.86)
BMI	Normal, *N* (%)	12 (40.0)	12 (31.6)	7 (50.0)	38 (39.6)
	Overweight, *N* (%)	9 (30.0)	12 (31.6)	3 (21.4)	35 (36.5)
	Obese, *N* (%)	9 (30.0)	14 (36.8)	4 (28.6)	23 (24.0)
Age at diagnosis, mean (SD)		53.5 (11.8)	47.5 (8.4)	58.8 (11.6)	51.7 (9.7)
Age at treatment initiation, mean (SD)		53.5 (11.8)	48.5 (8.2)	59.8 (11.1)	52.3 (9.7)
		N (%)	N (%)	N (%)	N (%)
Grade	1	0 (0)	1 (2.7)	0 (0)	0 (0)
	2	16 (53.3)	19 (51.4)	8 (57.1)	37 (38.5)
	3	14 (46.7)	17 (45.9)	6 (42.9)	59 (61.5)
Stage	IA	7 (23.3)	20 (52.6)	7 (50)	22 (22.9)
	IB	3 (10)	1 (2.6)	0 (0)	5 (5.2)
	IIA	12 (40)	12 (31.6)	4 (28.6)	20 (20.8)
	IIB	3 (10)	3 (7.9)	1 (7.1)	22 (22.9)
	IIIA	3 (10)	1 (2.6)	1 (7.1)	17 (17.7)
	IIIB	1 (3.3)	1 (2.6)	1 (7.1)	3 (3.1)
	IIIC	1 (3.3)	0 (0)	0 (0)	7 (7.3)
T	I	11 (36.7)	21 (55.3)	9 (64.3)	48 (50)
	II	16 (53.3)	15 (39.5)	4 (28.6)	45 (46.9)
	III	2 (6.7)	1 (2.6)	0 (0)	1 (1)
	IV	1 (3.3)	1 (2.6)	1 (7.1)	2 (2.1)
N	0	19 (63.3)	30 (78.9)	8 (61.5)	31 (33)
	1	8 (26.7)	8 (21.1)	4 (30.8)	37 (39.4)
	2	2 (6.7)	0 (0)	1 (7.7)	19 (20.2)
	3	1 (3.3)	0 (0)	0 (0)	7 (7.4)
Ki-67%, mean (SD)		44.6 (25.2)	30.8 (19.5)	35 (24.8)	38.4 (25.5)
Estrogen receptors	−	7 (23.3)	12 (31.6)	6 (42.9)	33 (34.4)
	+	3 (10)	3 (7.9)	1 (7.1)	8 (8.3)
	++	7 (23.3)	11 (28.9)	2 (14.3)	25 (26)
	+++	13 (43.3)	12 (31.6)	5 (35.7)	30 (31.3)
Progesteron receptors	−	9 (30)	15 (39.5)	11 (78.6)	57 (59.4)
	+	5 (16.7)	8 (21.1)	0 (0)	11 (11.5)
	++	6 (20)	8 (21.1)	1 (7.1)	13 (13.5)
	+++	10 (33.3)	7 (18.4)	2 (14.3)	15 (15.6)
HER-2	+	1 (3.3)	1 (2.6)	0 (0)	0 (0)
	++	2 (6.7)	9 (23.7)	3 (21.4)	12 (12.5)
	+++	27 (90)	28 (73.7)	11 (78.6)	84 (87.5)
FISH, mean (SD)		6.5 (2.4)	5.3 (2.4)	5.4 (2.6)	5.1 (2.5)
Hormone therapy	Yes	26 (86.7)	29 (76.3)	8 (57.1)	65 (67.7)
	No	4 (13.3)	9 (23.7)	6 (42.9)	31 (32.3)
Radiotherapy	Yes	29 (96.7)	31 (81.6)	8 (57.1)	79 (82.3)
	No	1 (3.3)	7 (18.4)	6 (42.9)	17 (17.7)

−, +, ++, +++: The degree of the Estrogen, Progesteron Receptor expression and HER-2 ranging from negative to strongly positive. 1,2,3: is the grading of the tumour. IA, IB, IIA, IIB etc: is the tumour staging based on Tumor-Node-Metastasis (TNM) classification.

**Table 4:** Treatment and test costs (€).

Parameters					The Three Subgroups of Trastuzumab Arms					
	Control (*N* = 30)		Trastuzumab (*N* = 148) (All Trastuzumab Arms)		Anthracyclines and Then Trastuzumab (*N* = 38)		No Anthracyclines and Trastuzumab (*N* = 14)		Anthracyclines, Taxanes, and Trastuzumab (*N* = 96)	
Costs (€)	Mean	SD	Mean	SD	Mean	SD	Mean	SD	Mean	SD
1st Chemo	2786.90	604.85	2632.91	905.46	3097.47	396.03	182.14	63.87	2806.43	452.88
2nd Chemo *	5105.47	238.88								
Trastuzumab **			38,069.95	6226.03	38,210.71	5499.80	37,240.37	6380.80	38,135.20	6520.39
Total cost	3978.18	1763.79	45,420.45	7182.79	413,08.19	5588.75	43,664.83	6672.79	47,304.25	7125.32

* Relates only to patients who did the 2nd chemotherapy (*N* = 7). ** The trastuzumab-related costs included costs of diagnostic tests, blood tests, the cost to determine HER2 status (immunohistochemistry (IHC) tests, FISH tests), medications and supportive medications, hospital cost when receiving the therapy, and cardiac monitoring costs.

**Table 5:** A cost-effectiveness analysis for 20 years and lifetime horizon scenarios.

Treatment	20 Years Horizon			Lifetime Horizon		
	Total Cost (€)	QALYs	ICER *	Total Cost (€)	QALYs	ICER *
Control (*N* = 30)	3978.18	1.70		3978.18	1.82	
Trastuzumab (*N* = 148) (all trastuzumab arms)	45,420.45	2.72	40,436.10	45,420.45	4.15	17,753.85
Control (*N* = 30)	3978.18	1.42		3978.18	1.51	
Anthracyclines and then trastuzumab (*N* = 38)	41,308.19	3.46	18,301.55	41,308.19	4.17	8954.97
No anthracyclines and trastuzumab (*N* = 14)	43,664.83	3.14	23,138.90	43,664.83	2.95	13,445.63
Anthracyclines, taxanes, and trastuzumab (*N* = 96)	47,304.25	3.21	24,254.73	47,304.25	3.30	13,111.44

* Incremental cost-effectiveness ratios (ICERs).

**Table 6:** Two scenarios of probabilistic sensitivity analysis for 20-year and lifetime horizons.

Treatment	% of Simulations Made it More Cost-Effective					
	20-Year Time Horizon			Lifetime Horizon		
	€20,000/QALY Gained	€40,000/QALY Gained	€60,000/QALY Gained	€20,000/QALY Gained	€40,000/QALY Gained	€60,000/QALY Gained
Control group vs. Trastuzumab (all trastuzumab arms)						
Without trastuzumab (control group) (*N* = 30)	99.0%	50.9%	17.4%	33.4%	3.4%	1%
Treatment with trastuzumab (*N* = 148)	1.0%	49.1%	82.6%	66.6%	96.6%	99%
Control group vs. three subgroups of trastuzumab arms						
Without trastuzumab (control group) (*N* = 30)	34.7%	2.3%	0.3%	3.2%	0.3%	0%
Anthracyclines and then trastuzumab (*N* = 38)	55.8%	89.1%	95.1%	95.5%	98.7%	99%
No anthracyclines and trastuzumab (*N* = 14)	5.5%	5.8%	3.4%	1%	0.9%	1%
Anthracyclines, taxanes, and trastuzumab (*N* = 96)	4%	2.8%	1.2%	0.3%	0.1%	0%

## Appendix A

**Table A1:** Sensitivity analysis based on all trastuzumab arms—one-way sensitivity analyses.

Variable	20-Year Horizon		Lifetime Horizon	
	ICER (€/QALY)		ICER (€/QALY)	
	−10%	+10%	−10%	+10%
Cost				
Total cost without trastuzumab	40,824.22	40,047.94	17,924.26	17,583.42
Total cost with trastuzumab	36,004.33	44,867.87	15,808.04	19,699.66
Discount rate	38,890.04	42,030.80	16,489.50	19,085.66
Utility weights				
EQ-5D For stable condition	45,037.52	36,687.76	19,720.14	16,144.12
EQ-5D For recurrence	40,375.13	40,497.25	17,756.21	17,751.48
EQ-5D For metastasis	40,409.33	40,462.91	17,756.64	17,751.06

Note. ±10%: + or – 10 percent change in the value of base scenario analysis, for each parameter. ICER: incremental cost-effectiveness ratios.

**Table A2:** Sensitivity analysis based on the three subgroups of trastuzumab arms—one-way sensitivity analyses for a 20-year horizon.

Variable	20 Years Horizon					
	ICER ^1^ (€/QALY)		ICER ^2^ (€/QALY)		ICER ^3^ (€/QALY)	
	−10%	+10%	−10%	+10%	−10%	+10%
Cost						
Total cost without trastuzumab	18,496.57	18,106.51	23,370.82	22,906.95	24,477.41	24,032.02
Total cost with anthracyclines and then trastuzumab	16,276.37	20,326.74	23,138.90	23,138.90	24,254.73	24,254.73
Total cost with no anthracyclines and trastuzumab	18,301.55	18,301.55	20,593.10	25,684.74	24,254.73	24,254.73
Total cost with anthracyclines, taxanes and trastuzumab	18,301.55	18,301.55	23,138.90	23,138.90	21,606.53	26,902.90
Discount rate	17,710.87	18,905.05	22,414.49	23,877.66	23,495.30	25,029.62
Utility weights						
EQ-5DFor stable condition	20,233.15	16,706.62	25,358.89	21,276.31	26,967.57	22,037.80
EQ-5D For recurrence	18,364.14	18,239.38	23,169.53	23,108.35	24,222.68	24,286.85
EQ-5D For metastasis	18,322.34	18,280.81	23,398.81	22,884.69	24,272.18	24,237.29

Note. ±10%: + or – 10 percent change in the value of base scenario analysis, for each parameter. ICER ^1^: incremental cost-effectiveness ratios for anthracyclines and then trastuzumab. ICER ^2^: incremental cost-effectiveness ratios for no anthracyclines and trastuzumab. ICER ^3^: incremental cost-effectiveness ratios for anthracyclines, taxanes, and trastuzumab.

**Table A3:** Sensitivity analysis based on the three subgroups of trastuzumab arms—one-way sensitivity analyses for a lifetime horizon.

				Lifetime Horizon			
Variable		ICER ^1^ (€/QALY)		ICER ^2^ (€/QALY)		ICER ^3^ (€/QALY)	
		−10%	+10%	−10%	+10%	−10%	+10%
Cost							
Total cost without trastuzumab		9050.39	8859.54	13,580.40	13,310.85	13,231.82	12,991.05
Total cost with anthracyclines and then trastuzumab		7964.05	9945.90	13,445.63	13,445.63	13,111.44	13,111.44
Total cost with no anthracyclines and trastuzumab		8954.97	8954.97	11,966.31	14,924.98	13,111.44	13,111.44
Total cost with anthracyclines, taxanes, and trastuzumab		8954.97	8954.97	13,445.63	13,445.63	11,679.90	14,542.97
**Discount rate**		8370.58	9563.48	12,649.46	14,267.28	12,316.13	13,934.89
**Utility weights**							
EQ-5D For stable condition		9898.65	8175.55	14,646.77	12,426.57	14,561.12	11,924.28
EQ-5D For recurrence		8986.22	8923.93	13,466.29	13,425.04	13,105.70	13,117.19
EQ-5D For metastasis		8965.70	8944.26	13,670.06	13,228.46	13,122.93	13,099.97

Note. ±10%: + or – 10 percent change in the value of base scenario analysis, for each parameter. ICER ^1^: incremental cost-effectiveness ratios for anthracyclines and then trastuzumab. ICER ^2^: incremental cost-effectiveness ratios for no anthracyclines and trastuzumab. ICER ^3^: incremental cost-effectiveness ratios for anthracyclines, taxanes, and trastuzumab.

## Appendix B

Linear regression analysis by the sequential integration/removal process (stepwise) was used to find independent factors related to the cost/QALY ratio, which resulted in dependency factors (β) and their standard errors (SE).

**Table A4:** Linear regression analysis.

All Trastuzumab Arms				
		β ^+^	SE ^++^	*p*
BSA (m^2^)		64,936.5	13,108.6	<0.001
The degree of positive expression of progesterone receptors		−5025.1	1575.1	0.002
Radiotherapy	No (ref.)			
	Yes	14,604.0	5196.9	0.006
The number of infiltrated lymph nodes (ΤΝΜ)		5095.7	2247.3	0.025
**Patients who received anthracyclines and then trastuzumab**				
The age at diagnosis		−1016.21	353.74	0.006
BMI		1490.76	575.28	0.012
**Patients who received no anthracyclines and then trastuzumab**				
Radiotherapy	No (ref.)			
	Yes	35,142.76	12,002.59	0.006
**Patients who received anthracyclines, taxanes, and trastuzumab**				
BSA (m^2^)		63,184.62	17,131.82	<0.001
The degree of positive expression of progesterone receptors		−5811.02	2099.99	0.007
Radiotherapy	No (ref.)			
	Yes	20,007.28	7839.48	0.012
The number of filtered lymph nodes (ΤΝΜ)		8363.22	2877.73	0.004

**^+^** Dependency factors (βs) and **^++^** their standard errors (SEs).
